# A night of sleep deprivation alters brain connectivity and affects specific executive functions

**DOI:** 10.1007/s10072-021-05437-2

**Published:** 2021-07-10

**Authors:** Matteo Pesoli, Rosaria Rucco, Marianna Liparoti, Anna Lardone, Giulia D’Aurizio, Roberta Minino, Emahnuel Troisi Lopez, Antonella Paccone, Carmine Granata, Giuseppe Curcio, Giuseppe Sorrentino, Laura Mandolesi, Pierpaolo Sorrentino

**Affiliations:** 1 Department of Motor Sciences and Wellness, University of Naples “Parthenope”, Naples, Italy; 2 Institute of Applied Sciences and Intelligent Systems, CNR, Pozzuoli, Italy; 3 Department of Social and Developmental Psychology, University of Rome “Sapienza”, Rome, Italy; 4 Department of Biotechnological and Applied Clinical Sciences, University of L’Aquila, L’Aquila, Italy; 5 Institute for Diagnosis and Cure Hermitage Capodimonte, Naples, Italy; 6 Department of Humanities Studies, University Federico II, Via Porta di Massa 1, 80133 Naples, Italy; 7 Institut de Neurosciences Des Systèmes, Aix-Marseille Université, Marseille, France

**Keywords:** Magnetoencephalography, Brain network topology, Cognitive function, Attention, Task switching

## Abstract

Sleep is a fundamental physiological process necessary for efficient cognitive functioning especially in relation to memory consolidation and executive functions, such as attentional and switching abilities. The lack of sleep strongly alters the connectivity of some resting-state networks, such as default mode network and attentional network. In this study, by means of magnetoencephalography (MEG) and specific cognitive tasks, we investigated how brain topology and cognitive functioning are affected by 24 h of sleep deprivation (SD). Thirty-two young men underwent resting-state MEG recording and evaluated in letter cancellation task (LCT) and task switching (TS) before and after SD. Results showed a worsening in the accuracy and speed of execution in the LCT and a reduction of reaction times in the TS, evidencing thus a worsening of attentional but not of switching abilities. Moreover, we observed that 24 h of SD induced large-scale rearrangements in the functional network. These findings evidence that 24 h of SD is able to alter brain connectivity and selectively affects cognitive domains which are under the control of different brain networks.

**Supplementary Information:**

The online version contains supplementary material available at 10.1007/s10072-021-05437-2.

## Introduction

Sleep is an active physiological process essential for well-being and for cognitive functioning. In fact, it promotes learning and memory as well as executive functions [1]. In this line, many electrophysiological studies have been evidenced that a good quality of sleep is associated with better performances in task assessment working memory and set-shifting abilities as well as in that related to planning, cognitive flexibility, and attention [2, 3]. Conversely, poor quality and quantity of sleep negatively affect mood and cognition [4]. Being an increasingly frequent condition, the effects of sleep deprivation (SD) are more and more studied. For example, EEG and magnetic resonance-based evidence indicate that sleep loss affects primarily the frontal lobes with serious repercussions on executive functions. The involvement of the frontal lobes is also documented by the evidence that sleep pressure (i.e., the felt urge to sleep) relates to increased theta power density mostly in these areas [5, 6] and by the reduction in fMRI signals in prefrontal pathways following SD [7]. Attention is one of the cognitive abilities most affected by SD. It underlies goal-directed behavior and deteriorates in a dose-dependent manner with prolonged wakefulness [8]. In addition, it has been reported that SD affects top-down but not bottom-up processing [9].

High-order cognitive functions, such as executive ones, need distributed coordinated activity across multiple, diffuse areas, being modulated by a “multifocal neural system” formed by multiple interacting areas [10, 11]. As a consequence, capturing the complex patterns of coordinated activity is necessary in order to explain them. To this regard, mathematical tools such as “Graph Theory” land themselves nicely to the study of the brain [12]. Under this framework, the brain is conceptualized as a complex system of interacting units, represented as nodes (brain areas), linked by edges (representing structural, functional, or causal interactions) [13]. This way, some topological features of the large-scale connections have been described consistently, such as “small-worldness” and “scale-freeness” [14, 15]. Such topological structure allows optimal and efficient communication under spatial and energetic constraints. Consequently, pathological conditions that disrupt this optimally tuned configuration are deleterious, either as excessive disconnections as in Alzheimer’s disease [16] or in terms of hyper-connectedness, as it occurs in epilepsy and amyotrophic lateral sclerosis [17, 18]. In fact, while disconnectedness hinders efficient communication, hyper-connectedness impairs brain flexibility [19].

The effects of SD on brain connectivity have been investigated by means of fMRI and EEG studies, reporting reduced connectivity within the dorsal attention network, the visual network, and especially the default mode network (DMN) [20, 21]. Prolonged wakefulness acts against the physiological decoupling between DMN and attentional networks [22], and this feature is associated with the worsening of vigilance and attention [23]. It has been observed that this impairment is related to the alteration of the topological changes in the attentional networks [24]. However, fMRI and EEG techniques have advantages and limitations [25]. In particular, fMRI offers optimal spatial resolution, but it is suboptimal in terms of temporal resolution. Furthermore, the estimation of functional connection in fMRI is done by correlating the amplitudes of the signals. Instead, EEG has optimal temporal resolution but poor spatial resolution, given that the electric fields are distorted by the layers surrounding the brain, so magnetoencephalography (MEG) is a useful neurophysiologic technique as it allows an optimal trade-off between spatial and temporal resolutions [26]. Considering the potential of MEG, we use this technique to study the brain topology after 24 h of SD in young adults. Based on source-reconstructed MEG signals, we built a functional network where the links represent synchronization between any two areas. To estimate synchronization, we choose the phase linearity measurement (PLM), a phase-based metric designed to be robust to noise and insensitive to volume conduction [27]. In order to avoid potential biases induced by different edge densities or average degree, we used the minimum spanning tree (MST), obtaining comparable networks [28].

To the best of our knowledge, this is the first MEG-based study on SD in young healthy adults, since most of the researches using this technique study SD in epileptic patients [29, 30]. In the present study, we investigated the effects of 24-h long SD on specific cognitive domains and topological changes. In particular, by means of specific cognitive tasks, we analyzed SD effects on selective attention and switching ability. Selective attention allows to focus exclusively on the characteristics of a target object, excluding any other distracting stimulus [31], while switching ability, involving inhibitory and executive control, allows to instantly readjust the behavior in front of different environmental demands, adapting thus the adapt behavior [32]. We hypothesized that SD modifies the organization of the brain network, as captured by modified functional topology, to a less efficient large-scale communication. We further hypothesized that such topological rearrangements would relate to an alteration of cognitive performance. To this aim, thirty-two young adults underwent MEG recordings and were evaluated in attentional and switching tasks before and after 24 h of SD. Observed topological changes were then correlated to cognitive performance.

## Material and methods

### Participants

Thirty-two young adult males were recruited (mean age ± SD, 24.84 ± 2.85 years). We excluded female participants from the sampling as the hormonal variations of the menstrual cycle influence brain connectivity [33, 34] and could represent a confounding variable (albeit controllable). All participants were right-handed, Italian speakers, and none of them had a history of medical, neurological, or psychiatric illness nor of medication or drug intake. The requirement of inclusion was normal sleep duration and no excessive daytime sleepiness. The quality and quantity of the participants usual sleep and daytime sleepiness were assessed by Pittsburgh Sleep Quality Index (PSQI) [35], the Epworth Sleepiness Scale (ESS) [36], and the Karolinska Sleep Diary (KSD) [37]. Subjects with scores less than 5 on the PSQI and less than 10 on the ESS were included in the study. From the third day before the beginning of the experimental procedure, each participant was required to maintain a regular sleep–wake cycle, and actigraphic recordings (wActiSleep-BT, ActiGraph-BT, Pensacola, Florida) were collected to control the subjects’ compliance. The intake of coffee, beverages containing stimulating active ingredients, and intense physical activity were prohibited starting 24 h before the experimental procedure, which was performed during the working week to avoid changes related to weekend activities. All participants gave their written informed consent. The study was approved by Ethical Committee of Psychological Research of the Department of Humanities of the University of Naples Federico II (n prot 11/2020) and was conducted in accordance with the Declaration of Helsinki.

### Procedure

The procedure was carried out by 4 participants at a time so that the night of SD was spent in the group. The experimental protocol included two sessions which took place at 09.00 a.m. at day 1 (T0) and 24 h later on day 2 (T1). In each session, the participants underwent MEG recordings at rest and immediately after they performed the Letter Cancellation Task (LCT) and the Task Switching Task (TS). During experimental sessions, participants were seated on a comfortable chair in a soundproof room. After the first session, the participants were free to return to their daily life activities. At 09.00 p.m. they returned to the laboratory to begin the SD under the experimenter’s supervision. To prevent them from falling asleep, they were allowed to take short walks outside the laboratory during the night. In both sessions, we assessed the perceived subjective state of sleepiness through the administration of the Karolinska Sleepiness Scale (KSS) [38], and the cognitive load by means of the NASA Task Load Index (NASA-TLX). Particularly, NASA-TLX is a multidimensional scale designed to obtain workload (i.e., the cost incurred by an individual to achieve a particular level of performance) estimates while performing a task. It consists of six subscales that represent somewhat independent clusters of variables (mental, physical, and temporal demands, frustration, effort, and performance) [39, 40].

### Cognitive assessment

All participants after MEG registration were taken to another room. For each test, the experimenter provided the instructions and left the room immediately, making sure the tests ran without distractions. The participants were seated on a comfortable chair and conducted the tests without distractions inside the well-lit and soundproofed room.

#### Letter cancellation task

The letter cancellation task [41] requires participants to search and mark sequentially (from left to right and from top to bottom), as fast and as accurately as possible, three target letters within a 36 × 50 matrix of capital letters (fonts: New York, “12”) printed on an A4 paper sheet. A maximum completion time of 5 min was allowed. Every target appeared 100 times in a random sequence; for each matrix, 300 hits were possible. Different parallel forms with different target letters were used in T0 and T1. Number of hits (as measures of accuracy) and number of rows completed (as index of speed) were considered dependent variables.

#### Task switching

In task-switching, two different tasks were performed in rapid succession and according to a random sequence of task presentation, so that the task to be executed might change from one trial to the next (“switch” trial), or be repeated (“repetition” trial). Task switches are usually slower and less accurate than task repetitions, and this difference is often referred to as the “switch cost” (SC). This cost is thought to reflect the time needed for the executive control processes to reconfigure the cognitive system for the execution of a new task [42] so that it can be considered an operational measure of the executive control [43].

All the participants were individually tested in a well-lit, sound-proof room. They were seated in front of a 15-in. computer monitor, at a distance of 50 cm, and at the beginning of each session, task instructions were both displayed on the screen and explained verbally by the experimenter, emphasizing the need for both accuracy (avoiding errors) and speed (minimize reaction times). In this study, the two tasks require deciding if a digit stimulus was odd or even (task A), or if it was greater or smaller than 5 (task B). In each trial of the two tasks, a cue (the “square” or “diamond” respectively) indicated the specific task (A or B) to perform on the subsequent target stimulus that appeared inside the cue. Experimental subjects used their left and right index fingers to provide their response: odd digits and digits smaller than 5 were mapped onto the left index finger response, whereas even digits and digits larger than 5 were mapped onto the right index finger response. The same two response keys on the computer keyboard (“A” for left and “L” for right index finger) were used for both tasks. Stimuli presentation and response recordings were managed to employ custom software (Superlab, version 4.0.4 for Windows, Cedrus Corporation).

Each participant initially performed a training session (2 blocks of 80 trials) followed by an experimental session consisting of 320 trials, arranged in 4 blocks of 80 trials each. We considered the task as learned when, during the training session, at least 85% of the correct response were achieved. On each trial, a cue was presented for 1000 ms, and then, it was followed by a target stimulus that remained on the monitor until the participant’s response. A schematic description of the present task-switching paradigm is reported in the “Supplementary information” section (S.I.1).

### MEG acquisition

The MEG system, developed by the National Research Council, Pozzuoli, Naples, at Institute of Applied Sciences and Intelligent Systems “E. Caianiello,” is equipped with 154 magnetometers and 9 reference sensors located on a helmet. MEG acquisition was performed as described in Jacini (2018) [44, 45]. The magnetic fields were recorded for 7 min, divided into two-time intervals of 3′ 30″.

To provide a directional estimate of the connectivity, the phase linearity measurement (PLM) was performed [27]. We excluded from the analysis the cerebellar regions, given the low reliability, leaving 90 regions encompassing the cerebral cortex and the basal ganglia. The obtained weighted adjacency matrix was used to reconstruct a brain network, where the 90 areas of the AAL atlas are represented as nodes, and the PLM values form the weighted edges. For each trial longer than 4 s and for each frequency band, through Kruskal’s algorithm, the minimum spanning tree (MST) was calculated [28].

Finally, global and nodal parameters were calculated. The global parameters included the diameter (*D*), defined as the longest shortest path of an MST, representing a measure of the ease of communication across a network; the leaf fraction (*L*), defined as the fraction of nodes with a degree equal to 1 (leaf), providing an indication of the integration of the network; the degree divergence (*K*), a measure of the broadness of the degree distribution, related to resilience against targeted attacks; and the tree hierarchy (Th), defined as the number of leaves over the maximal betweenness centrality, meant to capture the optimal trade-off between network integration and resiliency to hub failure. The nodal parameters included the degree (k), defined as the number of connection incidents on a given node, and the betweenness centrally (BC) described as the number of the shortest paths passing through a given node over the total of the shortest paths of the network. Before moving to the statistical analysis, all the metrics were averaged across epochs in order to obtain one value for the subject [46]. A pipeline of the processing MEG data is illustrated in S.I.2.

### MRI acquisition

Magnetic resonance imaging (MRI) data were used for the source reconstruction. Based on an MRI, the volume conduction model proposed by Nolte was considered and the Linearity Constrained Minimum Variance (LCMV) beamformer was implemented to reconstruct time series related to the centroids of 116 regions of interest (ROIs), derived from the Automated Anatomical Labeling (AAL) atlas [44]. MRI images of thirty-two young adult males were acquired on a 1.5-T Signa Explorer scanner equipped with an 8-channel parallel head coil (General Electric Healthcare, Milwaukee, WI, USA). MR scans were acquired after the end of the SD protocol. In particular, three-dimensional T1-weighted images (gradient-echo sequence Inversion Recovery prepared Fast Spoiled Gradient Recalled-echo, time repetition = 8.216 ms, TI = 450 ms, TE = 3.08 ms, flip angle = 12, voxel size = 1 × 1 × 1.2 mm1; matrix = 256 × 256) were acquired.

### Statistical analysis

In order to assess the potential variations of the participants’ executive performance during the two experimental sessions (T0 vs T1), and to assess how both sleep deprivation and the consequent increase in sleepiness can affect their performance, we run two different statistical analysis, respectively two-factor repeated-measures ANOVA, with time and trial as within factor, and paired *t* test.

In particular, with regards to the TS, median reaction times (in ms; median RT) to both repetition and switch trials, and angular transformations of the proportion of errors resulting from the two experimental sessions, were submitted to two-factor repeated-measures ANOVA. SC and all dependent variables obtained from letter cancellation task (LCT) (number of hits and number of rows completed) were analyzed through paired *t* test. SCs were computed as the difference between median switch RT and median repetition RT. Proportions of errors (EP) were computed by including both incorrect and missing responses. Before statistical analysis, this variable was submitted to an angular transformation, *y* = arcsen [sqr(*p*)], where sqr(*p*) is the square root of the proportion. All statistical analyses were performed using IBM SPSS Statistics for Macintosh, version 25.0 (IBM Corp., Armonk, NY, USA).

With regard to the topological data, statistical analysis was performed in Matlab (Mathworks®, version R2018b). Non parametric Wilcoxon test was performed to compare T0 and T1 in all frequency bands; all *p* values were corrected for multiple comparison using the false discovery rate (FDR).

Subsequently, the Pearson’s correlation index was used to find possible correlations between topological data and behavioral performances. We calculated the difference of the values of all the variables between T1 and T0. Therefore, the correlation analysis was carried out between the differences in topological parameters and the differences in scores on cognitive tests (LCT, TS) and subjective evaluation (KSS, NASA-TLX). Alpha level was fixed at 0.05.

## Results

### Cognitive assessment

#### Letter cancellation task

**Fig. 1:**
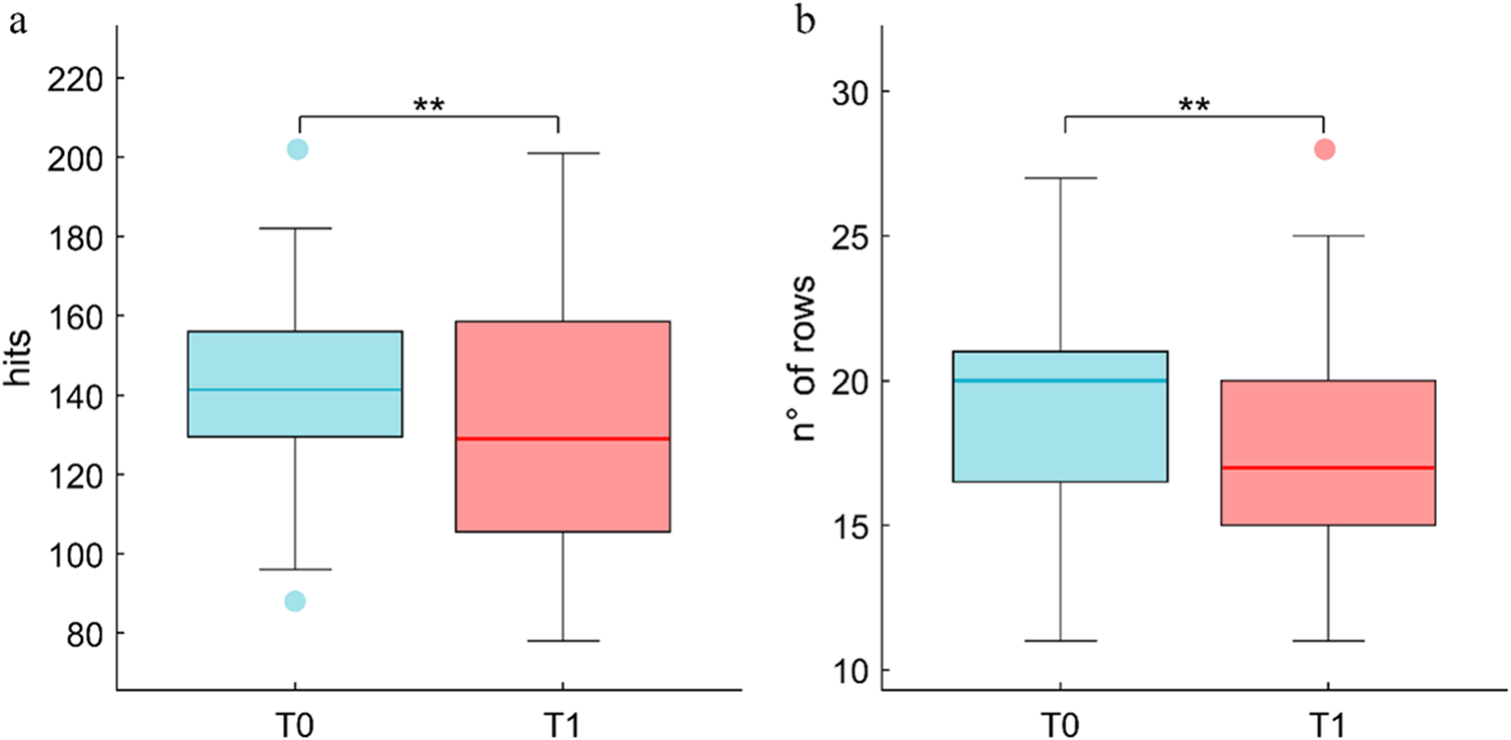
Letter cancellation test (LCT). Performance in LCT show the worsening of attentional funciton: accuracy (Fig. 1a) and speed (Fig. 1b) were reduced in T1 compared to T0. ** = p < .01

With regards the accuracy, represented by the number of hits, a better performance (*t*_31_ = 2.8; *p* = 0.009) at T0 (mean ± SE = 143.03 ± 4.32) than at T1 (mean ± SE = 132.34 ± 6.02) was demonstrated (Fig. 1a). As concern the speed, expressed as the number of inspected rows within the task time limit, a statistically significant difference was apparent between the two experimental sessions (*t*_31_ = 3.2; *p* = 0.003), as the subjects were faster in performing the test during T0 (mean ± SE = 19.12 ± 0.63) with respect to T1 (mean ± SE = 17.75 ± 0.7) (Fig. 1b).

#### Task switching

In task switching (TS), reaction times (RT), error proportion (EP), and switch cost (SC) (as measure of executive control) were assessed.

**Fig. 2:**
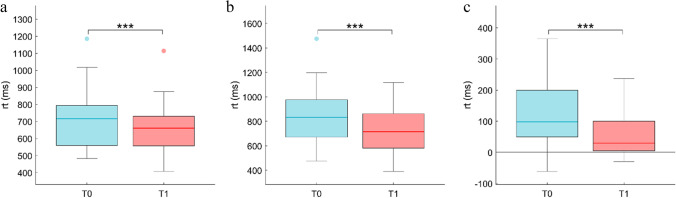
Task switching (TS). TS shows a reduction in reaction time (rt) both in repetition trial (RepT; Fig. 2a) and switch trial (SwT; Fig. 2b). Even the switch cost (SC; Fig. 2c) was reduced after 24 h of sleep deprivation. *** = *p* < .001

A statically significant effect of time on RT (*F*_1,31_ = 15.82; *p* < 0.0001; ηp2 = 0.32) was observed, indicating, regardless of the trial, higher RT at T0 (mean ± SE = 775.61 ± 36.22) than at T1 (mean ± SE = 690.67 ± 28.32). There was also a statically significant effect of the trial on RT (*F*_1,31_ = 39.4; *p* < 0.0001; ηp2 = 0.54), revealing a faster RT in repetition trials (mean ± SE = 689.2 ± 28.34) than in switch trials (mean ± SE = 777.09 ± 36.09). Finally, a statically significant interaction effect was found between time and trial (*F*_1,31_ = 12.74; *p* = 0.001; ηp2 = 0.28); repetition trials, T0 (mean ± SE) = 715.32 ± 30.35; T1 (mean ± SE) = 663.07 ± 25.83 (Fig. 2a); switch trials, T0 (mean ± SE) = 835.9 ± 38.87; T1 (mean ± SE) = 718.28 ± 30.22 (Fig. 2b). With regards to EP, a statically significant effect of trial on EP (*F*_1,31_ = 8.79; *p* = 0.006; ηp2 = 0.21) was showed, indicating, regardless of the time, higher accuracy during the “repetition trials” (mean ± SE = 1.49 ± 0.01) with respect the “switch trials” (mean ± SE = 1.44 ± 0.02). No other significant effects were observed. Finally, significantly higher SC (*t*_31_ = 3.57; *p* = 0.001) at T0 (mean ± SE = 120.57 ± 18.87) than at T1 (mean ± SE = 55.2 ± 11.73) was observed (Fig. 2c).

In order to evaluate a possible learning effect, before the first experimental session, a training session was performed and a comparison between the second block of the training and the first block of the test was performed. Results showed a reduction of RT in switch trials (*t*_31_ = 3.6; *p* < 0.001; training (mean ± SE) = 1062.3 ± 60.2; test (mean ± SE) = 860.9 ± 58.7) and switch cost (*t*_31_ = 2.5; *p* < 0.05; training (mean ± SE) = 261.2 ± 36.6; test (mean ± SE) = 133.8 ± 37.8). However, no significant difference in EP (indicating the achievement of maximum performance during the trial session, before the test) was evident.

### Subjective evaluations

**Fig. 3:**
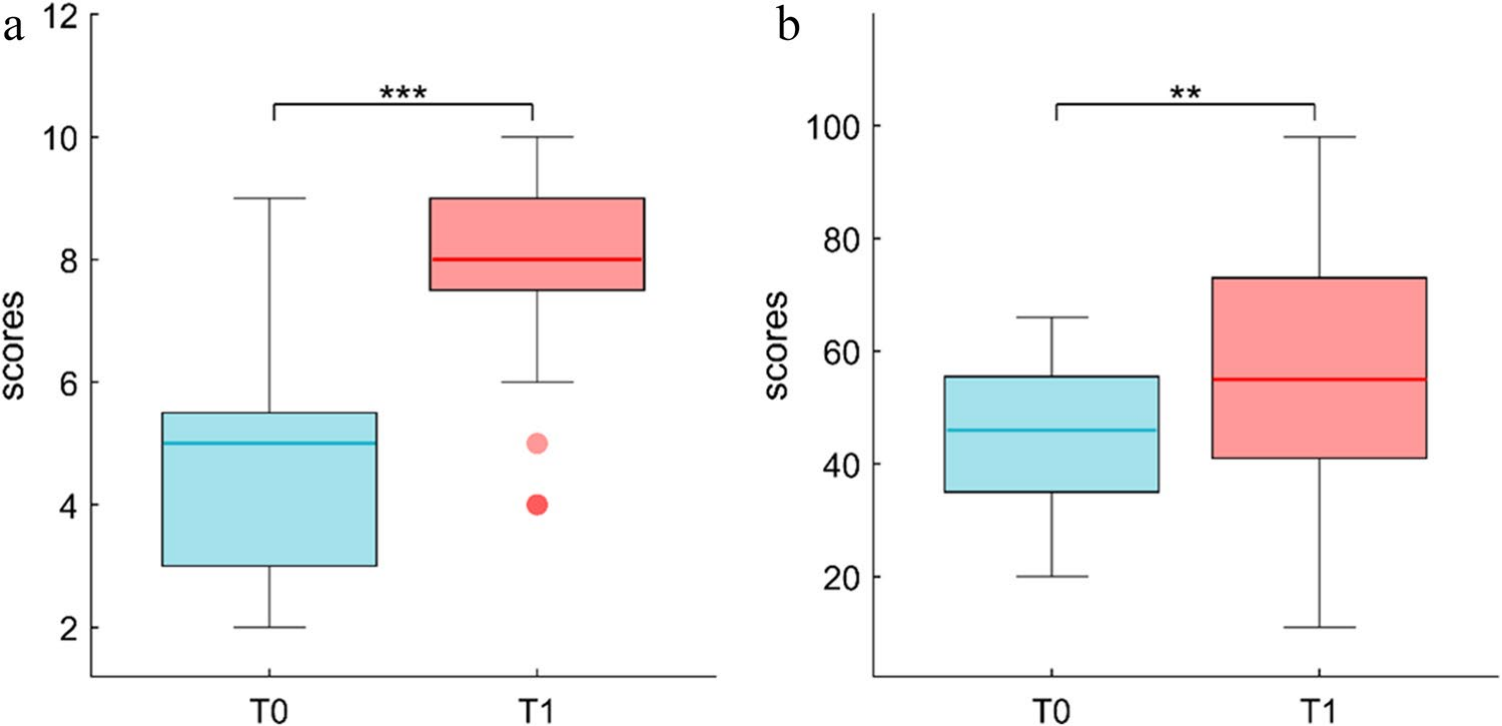
Subjective evaluation. Participants reported a significant increase in the subjective level of sleepiness (KSS; Fig. 3a) and perceived cognitive load (NASA-TLX; Fig. 3b) in T1 compared to T0. ** = *p* < .01, *** = *p* < .001

KSS results showed an increased level of sleepiness after 24 h of sleep deprivation (Fig. 3a; mean ± se; T0: 4.65 ± 0.32, T1:7.87 ± 0.25; *p* < 0.001), and the NASA-TLX index was increased in T1 as compared to T0 (Fig. 3b; mean ± se; T0: 43.75 ± 2.24, T1:54.03 ± 3.72; *p* < 0.01).

### Topological analysis

**Fig. 4:**
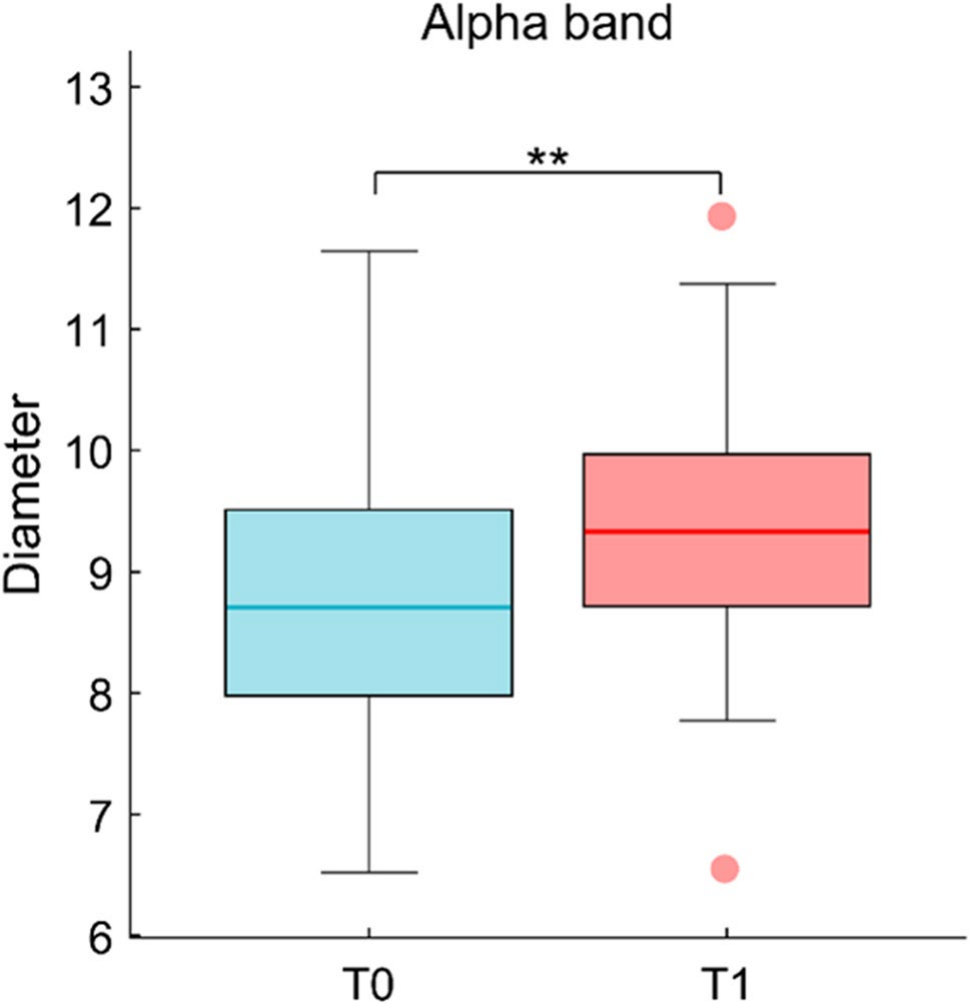
Topological analysis. Diameter. Statistical comparison between T0 and T1 shows a significant increase in diameter in alpha band after 24 h of sleep deprivation. ** = *p*_fdr_ < .01

The analysis for the global parameters showed an increased diameter in the alpha band (Fig. 4; median ± IQR; T0: 8.7 ± 1,48; T1: 9.33, 1,17; *p*_fdr_ < 0.01). No other global (leaf fraction, degree divergence, eccentricity, and three hierarchy) nor nodal (degree, betweenness centrality) parameters were different between the two conditions.

### Brain topology, performance, and sleepiness

**Fig. 5:**
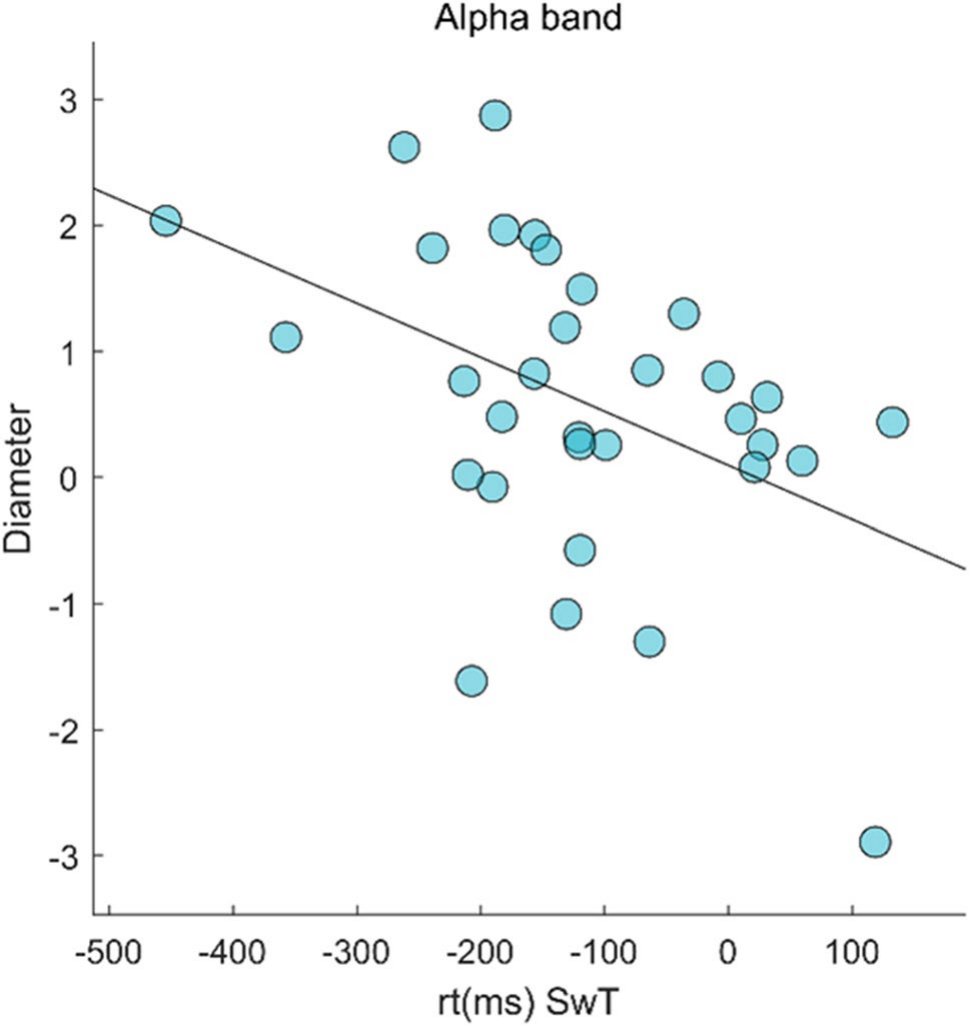
Correlation analysis. Pearson’s correlation between diameter in alpha band and switch trial reaction time

In order to evaluate a possible relationship between network rearrangements and cognitive functioning, we carried out a correlation analysis between topological parameters, test performance, and subjective evaluations. Specifically, Pearson’s correlation analysis showed a reverse correlation between the diameter in the alpha band and a switch trial reaction time (rt SwT) (Fig. 5; *p*_fdr_ = 0.03, *r* =  − 0.447). The letter cancellation task (LCT) performance, as well as the KSS and NASA-TLX scores, was not correlated with topological data.

## Discussion

In the present study, we set out to test the hypothesis that SD causes functional rearrangements in the brain so as to impair optimal communication, and that such rearrangements relate to changes in specific cognitive tasks, especially the ones requiring sustained attention. As we expected, after 24 h of SD, the participants reported increased subjective sleepiness, as seen from the scores of the KSS. SD negatively influenced attentional functions as evident from the worsening in the speed and accuracy of the LCT. Our data are in line with Casagrande [41] showing that the effect of SD on cognitive performance is prominent in tasks requiring high attentional load, and several authors reported that attention is among the first functions to be affected by SD [23]. Given the nature of the task, the attention deficit was possibly due to the weakening of the top-down processing.

A counterintuitive scenario was observed in the task switching (TS) where performance appeared slower at T0 than at T1, as shown by both higher RTs and SCs. Coherently, also, SCs appeared to be reduced as a consequence of SD. This unexpected effect of a single night of sleep loss cannot be ascribed to data instability. In fact, as an indirect measure of data goodness, statistical comparison of RT and EP at task switching highlighted faster RTs and reduced EP in repetition than switch trials, as expected on the light of previous literature [43], and this trend was confirmed in both pre- and post-sleep deprivation sessions. The unexpected improvement of the reaction times could lead erroneously to the conclusion that the switching ability improves following the SD. However, the number of errors (a fundamental parameter to verify the learning of a task) remained unchanged between the two sessions, so it cannot be considered that the performance had improved in absolute terms. The hypothesis that these results may be due to a learning effect between the two sessions does not appear to be the most likely, as all participants reached the maximum level of performance during the training phase. In fact, the comparison between the second block of the training phase and the first block of the experimental session at T0 revealed the absence of differences in the number of errors, confirming thus that the task had been fully learned in the training. If on the one hand the test was found to be reliable, we cannot ignore the context in which the entire experimental procedure was carried out. An important role could have been played by the social component of the experimental setting as the SD was carried out in groups of 4 participants at a time. As known, most of the studies use forced wakefulness protocols to evaluate the effects of SD on cognitions in participants who are not in social conditions. Multiple brain systems are affected differently based on the sleep deprivation model used. Deurveilher [47] showed that a condition of “voluntary” deprivation characterized by social interactions and exploration of the environment caused greater activation of the cholinergic pathways in the basal forebrain compared to a “forced” deprivation characterized by simple sensory stimulation. In this way, the increased activation supported the motivation to stay awake by attenuating the signs of SD. Similarly, the social interaction experienced in our experimental setting may have contributed to the result obtained in task switching.

The topological analysis showed that after SD the brain network undergoes rearrangements at the global level. Global metrics allow to characterize the widespread remodeling of large-scale brain activity. We found increased diameter after SD in the alpha band. The diameter is defined as the longest shortest path of a graph [28]. A network characterized by a small diameter presents a functional configuration with a few central nodes mediating long-range communication [48]. This arrangement is advantageous as it allows efficient communication throughout the entire network, at the cost of increased risk of node overload [49]. Conversely, a higher diameter implies a less compact topology with, on average, longer paths to go between any two nodes. This implies that the relative importance of nodes is more homogeneous [50]. Given the redistribution of the workload across the brain network, the node overload risk might be reduced. One possible interpretation of the increase in diameter that we observed would be that this topological rearrangement is in place to cope with cognitive demands after the SD, redistributing the computational load across the network. In fact, the inverse correlation between the diameter and the reaction times in switch trials of TS showed that topological rearrangements are related to cognitive performance. In particular, the sleep-deprived subjects have a less integrated topology and shorter reaction time. Hence, topological change allows to perform the task faster, specifically in switching trials (the most cognitively demanding trial). However, caution should be used when interpreting the data, given that the brain measures are made at rest and not during the execution of the task.

It is well known that a correct balance between integration and segregation of activity is relevant [51]. After 24 h of prolonged wakefulness, the brain networks appeared to have modified its overall integration. The correct balance between integration and segregation seems to be modulated by neurotransmitter-specific pathways. More specifically, cholinergic neurons have a strong influence in promoting segregation. Cholinergic tone is known to be modulated by wakefulness via the inhibition induced by adenosine (AD) on acetyilcholine (ACh) [52]. In fact, during SD, the increase in AD causes a modulation of the cholinergic system that, from the basal forebrain, project widely towards subcortical and cortical structures including the visual, somatosensory, and prefrontal cortex, controlling selective visual attention [53]. Even noradrenaline (NA) influences the network topology. Noradrenergic projections from the locus coeruleus are widespread to the whole brain and supports network integration [52]. Furthermore, high levels of NA have been associated with the ability to shift between tasks [54] and other higher order functions such as cognitive control. However, it is surprising that after 24 h of SD the participants performed the TS faster. Our interpretation is that this type of task is more engaging: in this case, the processing of information is externally driven through a bottom-up mechanism. In fact, selective damage to top-down versus bottom-up mechanisms after sleep deprivation has been demonstrated by Gevers [9]. In fact, the SD influences the attention and cognitive control required by the two tasks in a different way. In line with our interpretation, Trujillo et al. (2009) found that 1 night of sleep deprivation impacts endogenously driven selective attention (top-down) more than exogenously driven (bottom-up) selective attention [55]. Our results showed that the brain network was able to support optimal performance by compensating through greater cognitive effort, as suggested by the increased cognitive load detected through the NASA-TLX test. Moreover, this finding in in according with Tomasko and collegues [56] that showed that SD increases the cognitive load of learning surgical procedures that were otherwise well performed. In addition, it has been observed that even the mood can be modulated by SD [57]. Although in this study, the mood of the participants was not completely assessed, frustration is nevertheless a parameter evaluated by NASA-TLX. Therefore, we can assume that the increased frustration in performing the tasks after SD acted as a motivating component to perform the TS. However, this latter interpretation remains purely speculative.

Given the present results, it should be noted that the study described has some limitations, such as the relatively small sample size. However, it has to be considered that our sample consists of young adults of similar age. This feature represents a great advantage because the human brain changes during one’s life span and analyzing the brains of individuals of the same age allows us to study in accurate way SD effects of brain topology at a delimited age. Therefore, for future studies, in addition to increasing the sample and investigating other age groups, it could be also interesting to study a female population to evaluate any gender differences on the SD effects. In fact, it is known that brain electrical activity (and therefore brain connectivity) is influenced differently by hormones in the various phases of the menstrual cycle [33, 34]. In addition, investigating other cognitive domains would help to understand how connectivity and cognitive functioning changes following SD. The focus could be extended to other components of executive functions and to memory processes, involving cortical and subcortical structures, such as the parietal cortex and hippocampus and are severely damaged by lack of sleep [58, 59].

In conclusion, the present study evidence that 24 h of SD affects the topology of the brain network making it less integrated. The less integrated brain network causes selective damage to the attentional but not switching abilities. This is probably related to the fact that the stimuli of the two different tasks are processed in different ways through bottom-up and top-down processing, which are affected differently by sleep deprivation.

The knowledge of the effects of SD on cognitive functions could be important in understanding how to control cases in which individuals are subjected to activities that affect sleep, such happens in shift workers.

## Supplementary Information

Below is the link to the electronic supplementary material.

Supplementary file1 (DOCX 235 KB)

## Data Availability

The data that support the findings of this study are available from the corresponding author upon request.
