# Supplementary material for: A night of sleep deprivation alters brain connectivity and affects specific executive functions
Source: Neurol Sci. 2021 Jul 10;43(2):1025–34. doi: 10.1007/s10072-021-05437-2 (PMC8789640; doi:10.1007/s10072-021-05437-2)
Supplement: Supplementary file 1 — Supplementary file1 (DOCX 235 KB) [file 10072_2021_5437_MOESM1_ESM.docx]

**Supplementary Information**

**S.I 1. Schematic representation of Task Switching.** The two tasks require deciding if a digit stimulus was odd or even (task A), or if it was greater or smaller than 5 (task B). In each trial of the two tasks, a cue (the “square” or “diamond” respectively) indicated the specific task (A or B) to perform on the subsequent target stimulus that appeared inside the cue. The “L” key (right index finger) of the keyboard key was used provide the response “even” or “larger than 5”, while the “A” key (left index finger) was used to respond “odd” or “less than 5”. Each participant initially performed a training session (1 block of 80 trials) followed by an experimental session consisting of 320 trials, arranged in 4 blocks of 80 trials each. On each trial, a cue was presented for 1000 ms, and then it was followed by a target stimulus.

**S.I 2.** **Data analysis pipeline: a)** MEG signal recorded by 154 sensors; **b)** noisy channels, blinking and cardiac artifact are removed to obtain the cleaned signals; **c)** cleaned signals; **d)** MRI and MEG sensors are coregistered; **e)** the source activity has been estimated using a beamforming algorithm: the activity on the sensor level is projected onto brain regions (based on the AAL atlas); **f)** connectivity matrix: raws and colums are brain areas,entries are PLM values ; **g)** brain network representation based on the MST.

***S.I 1 Schematic representation of Task Switching.****.*


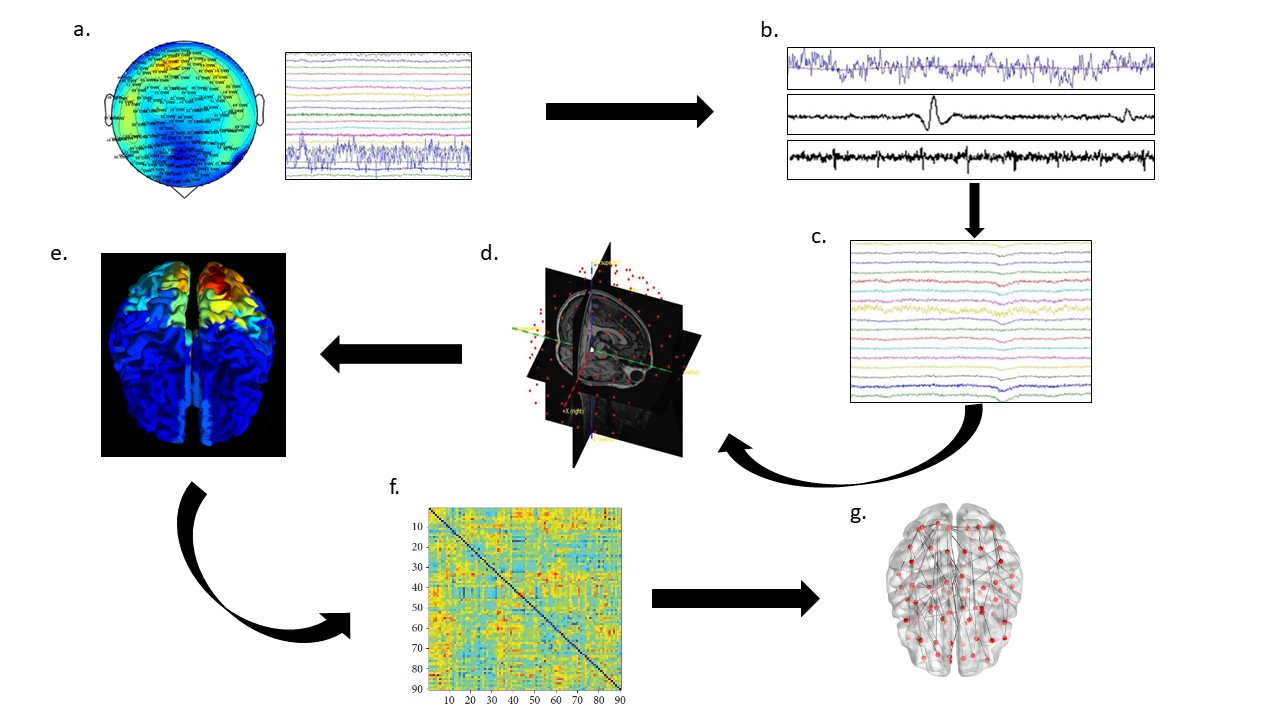


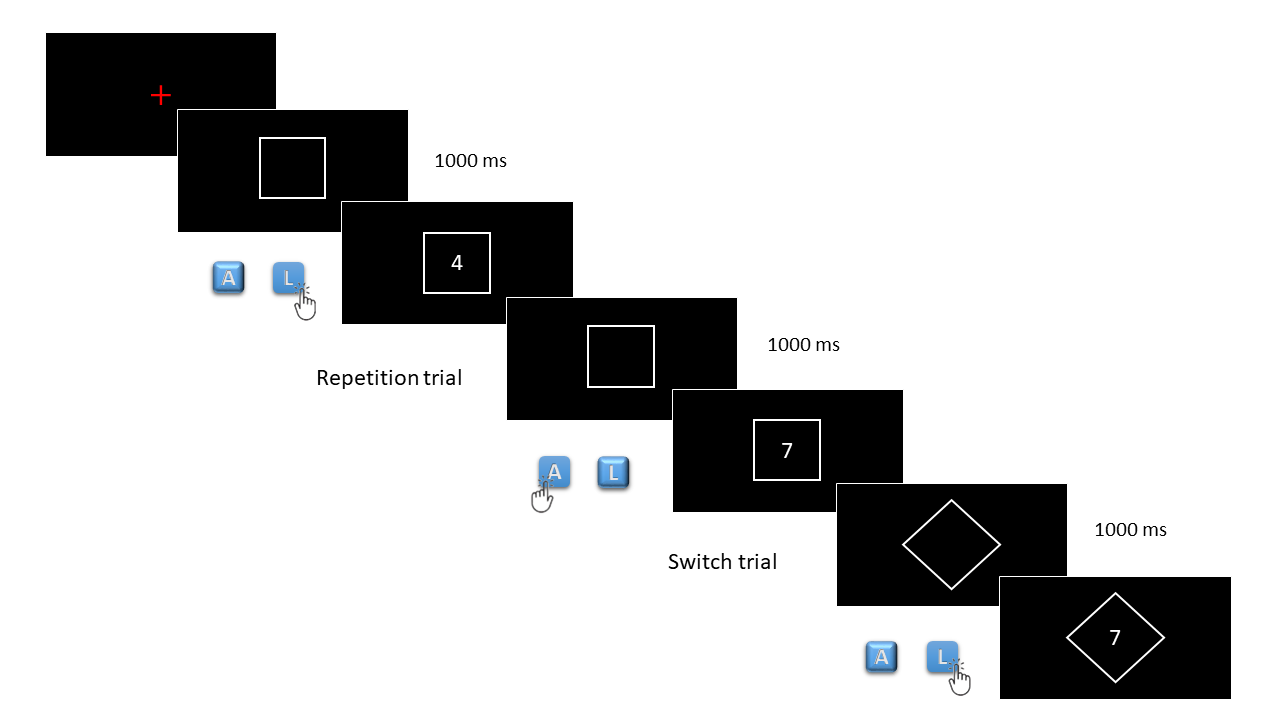
***S.I 2 Data analysis pipeline***
